# Waste Reduction Behaviors at Home, at Work, and on Holiday: What Influences Behavioral Consistency Across Contexts?

**DOI:** 10.3389/fpsyg.2018.02447

**Published:** 2018-12-06

**Authors:** Lorraine E. Whitmarsh, Paul Haggar, Merryn Thomas

**Affiliations:** School of Psychology, Cardiff University, Cardiff, United Kingdom

**Keywords:** recycling, theory of planned behavior, habits, spillover, waste reduction

## Abstract

Demand for materials is increasing, along with the environmental damage associated with material extraction, processing transport and waste management. While many people state they recycle at home, adoption of sustainable waste practices in the workplace and other contexts (particularly, on holiday) is often lower. Understanding how to promote more sustainable behaviors (including, but also going beyond, recycling) across a range of contexts remains a key challenge for policy-makers and researchers. The Theory of Planned Behavior (TPB) has been applied to a range of environmentally-friendly behaviors but the relative importance of the model's predictors has not yet been explored across a range of contexts. Here, we test the TPB across workplace (laboratory and office), home and holiday contexts, and examine whether consistency across contexts is a function of pro-environmental identity. Following ten semi-structured interviews, we undertook an online survey with laboratory workers (primarily in the UK; N = 213) to examine the predictors of recycling and waste reduction habits across these contexts. Interview findings indicate a range of motivations and barriers to recycling in the workplace, and inconsistency across home and work behaviors. Expanding the focus to include holiday as well as workplace and home contexts, our survey analysis shows that the proportion of waste recycled in the home is higher (67%) than in the workplace (39%) and on holiday (38%). Further, the TPB explained around twice as much variance in home recycling compared to work or holiday recycling; but overall did not provide a good explanation for recycling. The study highlights the importance of both contextual (e.g., facilities) and individual (e.g., identity) factors in shaping waste behaviors. We find significant correlations amongst different waste reduction behaviors within and between contexts, though within-context (e.g., home) behaviors are generally more strongly related. Future research should move beyond the TPB to expand the range of contextual (e.g., organizational) factors explored in contexts beyond the home, including workplace and holiday contexts. Given the different drivers-of and barriers-to waste reduction within and between contexts, a range of interventions will be required to promote recycling, reduction and reuse behaviors across these contexts.

## Introduction

### Waste Reduction Behaviors

Demand for materials is increasing, along with the environmental damage associated with material extraction, processing transport and waste management (Allwood et al., 2011). According to the “waste hierarchy” (reduce, reuse, recycle), which is the product lifecycle approach underpinning European legislation on waste (European Commission, 2014), the most effective means of reducing waste is to prevent waste in the first place (e.g., avoiding products with excessive packaging; consuming fewer products), followed by reusing or finding new uses for items, while recycling is the least effective strategy for reducing waste. While public awareness of waste-related problems (e.g., marine pollution) is growing (e.g., Hartley et al., 2018) and recycling rates are increasing in many countries (Eurostat, 2018), there has been less progress in reduce and reuse behaviors (Whitmarsh et al., 2011). For example, while only 3% of the UK public say they never recycle, this rises to 15% who never buy products with less packaging, and 30% who never avoid buying new things (Whitmarsh et al., 2017). Consequently, much waste continues to be generated and is often sent to landfill or for incineration (e.g., DEFRA, 2016).

While businesses and governments need to play a part in reducing waste, a significant role can also be played by individuals across the various contexts in which they consume and use materials. Little is known, however, about the predictors of waste reduction behaviors in different settings (e.g., home, workplace) or indeed how consistent individuals are across settings in this respect. Recycling research, though, suggests there is likely to be significant variation across contexts; for example, between the workplace and home (Tudor et al., 2008). Understanding how to promote more sustainable behaviors (including, but also going beyond, recycling) across a range of contexts remains a key challenge for policy-makers and researchers.

This paper aims to expand the behavioral and situational scope of waste reduction behavior research, which has largely focussed on recycling in the domestic context. We explore behavior at all levels of the waste hierarchy, including reduction, reuse and recycling behaviors; and we also examine these behaviors across three different contexts: home, workplace, and holiday.

### Influences on Waste-Reduction Behaviors

Recycling at home has been well-studied, and is influenced by both individual and contextual factors (Oskamp et al., 1991; Varotto and Spagnolli, 2017). Specifically, attitudes, knowledge, norms, demographics, habits and situational factors (e.g., collection frequency, recycling bin provision) have been shown to predict recycling behavior (e.g., Barr et al., 2003). Older, wealthier, more educated people and women have been shown to recycle more, while knowledge about environmental issues also predicts recycling behavior - particularly knowledge about recyclable materials, programmes and the location of recycling facilities (Geller et al., 1982; Vining and Ebreo, 1990; Schultz et al., 1995; Barr et al., 2003; Thomas and Sharp, 2013). Similarly, pro-environmental values or identity have also been shown to predict recycling behavior (Schultz et al., 1995; Whitmarsh, 2009; Huffman et al., 2014), particularly in the presence of recycling facilities (Derksen and Gartrell, 1993); indeed, having a kerbside recycling collection and other contextual factors (e.g., having space at home to store recycling) are typically the strongest predictors of recycling behavior (De Young, 1989; Barr et al., 2003; Varotto and Spagnolli, 2017). As recycling facilities have been expanded over recent decades, recycling has become easier and more normative. Both descriptive and injunctive social norms (i.e., perceptions of what most people are doing and what one ought to do, respectively) have increased amongst many societies, and in turn positively influenced recycling uptake (Thomas and Sharp, 2013). Consistent with this, interventions using social norms (coupled with psychological dissonance processes) have been found to encourage recycling behavior, with those making public commitments to recycle more likely to do so than those given information (Pardini and Katzev, 1984; cf. Bratt, 1999). Similarly, being asked to recycle by a local resident (“block leader”) has been shown to increase perceived social norms as well as personal norms (personal obligation) to recycle (Hopper and Nielsen, 1991). Habit has also been shown to predict recycling behavior (Carrus et al., 2008) as recycling has increasingly become part of domestic routines (Thomas and Sharp, 2013).

Somewhat less is known about what predicts other waste reduction behaviors, including prevention and reuse, although studies exploring these practices similarly suggest both psychological and contextual (e.g., socio-demographic) factors are relevant. For example, UK research found that those with higher education, altruistic values, and pro-environmental identity are more likely to buy products with less packaging; while younger, more educated and lower income people, and those with altruistic values and pro-environmental identity were more likely to avoid buying new things (Whitmarsh et al., 2017). Interventions to encourage waste reduction (beyond recycling) include financial measures, such as carrier bag charging and “pay-per-bin” schemes (i.e., local councils charge residents for each refuse bin filled), which have been found to be effective (Gardner and Stern, 1996; Poortinga et al., 2013). This indicates a lack of motivation to reduce waste rather than primarily structural impediments to waste reduction.

Similarly, relatively little work has explored waste reduction behaviors beyond the domestic context. Tudor et al.'s (2007) study of UK hospital employees' waste behaviors found personal beliefs about the benefits of recycling were the main predictor of recycling behavior, and concluded that the Theory of Planned Behavior is applicable in a workplace context. By contrast, Holland et al. (2006) conducted a workplace intervention (in offices of a Dutch telecoms company) to encourage recycling, and found that behavioral intentions were a poor predictor of recycling behaviors, whereas habits and recycling facilities were key predictors. These divergent findings from very different organizational contexts highlight the need for further research into the predictors of recycling and other waste behaviors in a workplace context, including exploring variation across workplace environments (offices, labs, factories, schools, etc.) with associated diverse forms of waste and waste management.

Similarly, little research exists on waste reduction behaviors on holiday. In general, waste reduction initiatives in tourist and hospitality industries tend to focus on change in organizational processes and staff behavior, while attempts to change visitors' behaviors are less common (Pirani and Arafat, 2014). The very limited work that has been done on the links between sustainable tourism and other contexts suggests that individuals are likely to do significantly less for the environment while on holiday than at home, at least partly due to reduced motivation (i.e., they want a break from all obligations, including pro-environmental ones; Barr et al., 2010; Cohen et al., 2013) but also due to social and structural impediments (e.g., social norms, cost of different travel modes; Randles and Mander, 2009). The exception to this may be eco-tourist resorts which actively encourage pro-environmental actions; one study found recycling levels were similar (around 40%) between home and resort, although this sample is likely have been more environmentally-committed than the general public (Lee and Moscardo, 2005). Amongst more diverse samples, where efforts are made by individuals to take their pro-environmental habits on holiday, these seem more often to be in respect of energy and water saving behaviors than other pro-environmental actions (Goldstein et al., 2008; Barr et al., 2010).

### Theory of Planned Behavior and Contextual Consistency

The Theory of Planned Behavior (TPB; Ajzen, 1991) has been applied to a range of environmentally-friendly behaviors, including waste reduction (Cheung et al., 1999; Kaiser et al., 2005). The TPB states that intentions predict behavior and that intentions are a function of social norms, attitudes, and perceived behavioral control. A study comparing the TPB with the Value-Belief-Norm (VBN) model of pro-environmental behavior found that the TPB predicted conservation behavior, including recycling, better than the VBN model (Kaiser et al., 2005). (The VBN model differs from the TPB in predicting behavior from personal moral norms rather than from behavioral intentions; personal norms, in turn, are predicted by beliefs about responsibility and environmental impact of behavior, and ultimately values). Indeed, many of the key influences on recycling behavior found in the studies described above map onto the TPB (e.g., perceived behavioral control reflects situational factors, such as availability of facilities), although other factors like identity, personal norm (sense of obligation) and knowledge, are also relevant for waste reduction behaviors but not explicitly part of the TPB. Similarly, given that waste-reduction behaviors can occur regularly and under similar circumstances (e.g., Holland et al., 2006) waste reduction could become a matter of habit, in which case this should also be taken into account, in addition to the TPB and other variables (Gardner, 2015).

However, the relative importance of the TPB variables and other predictors of waste reduction has not yet been explored across a range of contexts. We know from habit research (Verplanken, 2018) that context cues much of our behavior, meaning that many of our actions are inconsistent across different times and places (Nash et al., 2017). Similarly, there may be different motivations and barriers operating in different contexts, such as home and the workplace. For example, financial benefits of domestic energy saving may not exist at work, and control over equipment may be lower at work (Leygue et al., 2017). Indeed, previous research has found that workplace pro-environmental behaviors (e.g., setting up a recycling scheme at work) did not tend to co-occur with domestic or consumer behaviors, like recycling, turning off lights and buying green products (Whitmarsh et al., 2017). Even when comparing the same behavior across different contexts, there may be little or no relationship: Littleford et al. (2014) compared two Council workplaces and found notable differences between them in adoption of energy-saving behaviors, due primarily to control factors (e.g., automated lighting). They also found limited relationships between workplace and home energy-saving behaviors, although these relationships were stronger in one of the workplaces, where there was more control over behavior. They concluded that “people behave more consistently across settings when they have greater control over their own behavior,” including physical and social control (p. 165).

The relationship between work and home behaviors may indicate “situational” spillover—i.e., adopting a behavior in one context leads to adoption of the same behavior in another (Nash et al., 2017); this is contrasted with “behavioral spillover” which is where one behavior leads to adoption of another behavior in the same context (Thøgersen, 1999). Littleford et al.'s (2014) work suggests that control may mediate situational spillover, and that material factors (i.e., using the same equipment at home and work) may also be a facilitator. Other work also suggests home-work spillover may be possible if there is organizational or social support in both environments (Rashid and Mohammad, 2011); or if one has a strong pro-environmental identity (Frezza et al., in press). Identity-mediated spillover appears to have been greatest attention in previous literature; based on identity theories (e.g., Breakwell, 2014), the assumption here is that individuals' psychological drive for self-consistency and self-continuity underpins the transfer of behavior across contexts. Previous work appears to assume that any situational spillover is more likely to originate from a home behavior and be carried—via identity, attitudes or some other psychological construct—to the workplace (Tudor et al., 2008; Young et al., 2015). However, workplace interventions may trigger spillover to the home context (Frezza et al., in press). For example, Andersson et al. (2012) found spillover to home waste behaviors from a workplace recycling scheme. To date, little work has explored spillover across contexts—such as home and workplace—and to our knowledge, no studies have examined spillover across multiple contexts (e.g., home-work-holiday). The current study is therefore the first to explore multiple waste behaviors across home, workplace, and holiday contexts in order to examine both behavioral and situational spillover.

### Aims and Hypotheses

The present study examines waste behaviors across three main contexts—workplace (including lab and office), home and holiday. The research has two aims. Firstly, we compare the influence on recycling of TPB variables, pro-environmental identity and relevant situational variables (e.g., recycling facilities, organizational waste policy) in each of these contexts. Second, we explore the extent to which individuals are consistent in their waste reduction behaviors (recycling, reduction and reuse) within and across contexts, and whether identity predicts cross-context consistency.

In relation to the first aim, we expected that TPB variables (attitudes, social norm, PBC), identity, habits, personal norms and contextual variables (e.g., recycling information, location of bins) will predict recycling behavior across contexts; based on previous literature (e.g., Varotto and Spagnolli, 2017), PBC and contextual factors are hypothesized to exert the strongest influence. In relation to the second aim, we hypothesized that relationships between behaviors would be stronger within contexts than between contexts, because of the importance of contextual factors in predicting waste reduction actions. Further, consistent with dominant spillover models (Truelove et al., 2014; Nash et al., 2017), we hypothesized that pro-environmental identity would explain consistency in behaviors across contexts.

## Methods

Since waste reduction behaviors have been little studied outside of the domestic context, we undertook initial qualitative research to explore the range of influences on recycling, reducing and reuse behaviors in order to inform a subsequent quantitative survey. This sequential mixed-methods approach offers the advantage that quantitative measures are relevant and contextually-grounded (Creswell, 1999). Furthermore, as well as informing survey content (e.g., wording of TPB items), the interviews provided valuable insights in their own right on waste reduction behaviors. This rich and detailed qualitative data source has been used to triangulate and elaborate on findings from the survey stage, for example shedding light on salient motivations for and barriers to recycling (first aim) and when/why waste reduction behavior across contexts is (in)consistent (second aim). Conversely, the survey enabled quantitative analysis of the prevalence and predictors of waste reduction behaviors suggested by the interviews in a larger and more diverse sample.

The study was approved by Cardiff University's School of Psychology research ethics committee. Written informed consent was obtained from interviewees; and survey participants clicked on the initial information and consent page of the survey to confirm their informed consent (the survey only started if they clicked consent).

We selected a laboratory setting to conduct the workplace component of the research. Laboratories generate considerable waste, much of which is not recycled or reused due to contamination or infection risks (Hossain et al., 2011). In addition, researchers working in laboratories often spend time in other workplace settings, such as offices. This makes laboratory workers interesting to study from a multi-context perspective: we can study their behavioral consistency between laboratory and office settings within the workplace, as well as across the three broader settings of workplace, home and holiday.

### Interviews

We conducted interviews with laboratory workers (*N* = 10) working at a UK university. They were at different career-stages in several disciplines (biosciences, engineering, earth sciences, medicine). A convenience sample was recruited from amongst the authors' contacts, ensuring a balance of gender, seniority and discipline. Interviews lasted for around 30 min, were audio recorded and thematically coded using an inductive approach (Braun and Clarke, 2006). Interviews were semi-structured and intended to elicit prevalence, drivers and barriers in respect of waste reduction behaviors at work, with a particular focus on labs. The interview schedule covered the following topics: types of waste generated and how they are dealt with; which items are (not) recycled/reused, and why (not); awareness of waste facilities and policies; colleagues' waste behaviors; responsibility and reasons for reducing waste; and what measures would encourage more recycling and reuse.

### Survey

#### Participants

Following this, an online survey was undertaken with laboratory workers (*N* = 213) to examine the predictors of recycling and waste reduction habits across the three contexts. Participants were recruited through academic email lists and snowballing (asking colleagues working in laboratories to send on to others). Table 1 shows the sample composition. Most participants were from the UK and were early-career researchers working in universities. Table 1 shows the sample composition. Participants were also asked ‘what proportion of your time at work do you spend in your lab (as opposed to an office or elsewhere)?’: a mean of 44% was recorded.

**Table 1:** Survey sample characteristics.

**Gender**	**%**	**Job role**	**%**
Female	54	Student/PhD, Postdoc or Researcher	59
Male	44	Academic Staff	24
Prefer not to say	2	Manager	6
		Other (e.g., technicians)	11
**Age**			
16–25	13	**Subject**	
26–35	43	Biological	38
36–45	26	Medical	24
46–55	13	Earth/Environmental	23
56–65	4	Chemical	11
Over 65	0	Engineering/Maths/Computing	5
Prefer not to say	1		
		**Organization**	
**Location**		University/HEI	83
Wales	62	Private-sector organization	7
England	19	Other public-funded research organization	4
Scotland	2	NGO/charity	2
N. Ireland	0	Other	4
Other	17		
		**Member of environmental organization**	
		Yes	23
		No	77

#### Measures

Dependent variables were measured as follows.

- Proportion of waste recycled: “Roughly what proportion of your waste at work (including in your office, lab, public work areas, etc.) do you recycle?” with response indicated on a percentage slider. The question was repeated for “at home” and “on your last holiday.”
- Materials recycled: “Now thinking specifically about your laboratory, which (if any) of the following items do you recycle?” Items listed were those shown in Figure 3; respondents checked any of these they recycled. The question was repeated for “other areas at work, besides your laboratory (e.g., your office, kitchen, corridors)?”; “at home;” and “on your last holiday.”
- Proportion of materials reused: “Roughly, what proportion of the things you use in your laboratory (e.g., gloves, petri dishes) do you reuse or repair (instead of throwing away)?” with response indicated on a percentage slider. The question was repeated for things used “at home.”
- Other reuse and reduce behaviors included (a) carrier bag reuse: “How often do you take your own bag(s) when you go shopping?;” and (b) “How often do you choose products without too much packaging?” both with a five-point response scale from “Always” (5) to “Never” (0).

TPB variables were measured as follows. All responses were made using a seven-point scale from Strongly disagree (1) to Strongly agree (7).

- Attitude (α_home_ = 0.80, α_lab_ = 0.71, α_holiday_ = 0.81) comprised five items (adapted for the three primary contexts of interest: home, lab and last holiday). Three items began “I believe that recycling *at home [lab waste, on my last holiday]* benefits [benefited]” and ended with: (1) “me,” (2) “my local area” and (3) “then environment,” respectively. The other two items were: (4) “Recycling *at home [lab waste, on holiday]* poses risks to me and my family [colleagues]” (reverse-scored); and (5) “I think recycling *at home [lab waste, on holiday]* is a good idea.”
- PBC (α_home_ = 0.83, α_lab_ = 0.69, α_holiday_ = 0.67) was measured with two or three items, depending on context, with wording adapted to context: Recycling *at home [lab waste, on my last holiday]* is [was] too much of a hassle to bother with (reverse-scored); I avoid [avoided] recycling *at home [in my lab, on my last holiday]* due to lack of time (reverse-scored); I recycle *at home* because there are facilities available that make this easy (home only).
- Social Norms (α_home_ = 0.68, α_lab_ = 0.87, α_holiday_ = 0.79) comprised two items again with context-relevant wording: Most of *my friends and family [colleagues]* recycle *at home [their lab waste, on holiday]; My friends and family [colleagues]* encourage me to recycle *at home [in the lab, on holiday]*.

Additional predictors included the following.

- Personal Norm was measured with one item: I feel a moral obligation to recycle *at home [my lab waste, on holiday]*, again with responses on a seven-point agreement scale.
- Knowledge (α_home_ = 0.61, α_lab_ = 0.54, α_holiday_ = 0.72) was measured with two items: I know a lot about which materials can [could] be recycled *at home [in my lab; on my last holiday];* I know [knew] where to deposit items for recycling *where I live [where I went on my last holiday, in my lab]*, again using a seven-point agreement scale.
- Habit was measured with the four-item Self-report behavioral automaticity index (SRBAI; α_home_ = 0.95, α_lab_ = 0.95, α_holiday_ = 0.98) across the three contexts: Recycling *in my laboratory [at home, on my last holiday]* … is something I do [did] automatically; is something I do [did] without thinking; I do [did] without having to consciously remember; I start [started] doing before I realize [realized] I was doing it. Responses were on a seven-point scale from Strongly disagree (1) to Strongly agree (7).
- Pro-Environmental Identity (α = 0.83) was measured with six items that include general pro-environmental and more specific waste-conscious identity statements (adapted from Whitmarsh et al., 2017): I consider myself to be environmentally-conscious; Being environmentally-friendly is an important part of who I am; I think of myself as someone who is very concerned about environmental issues; I would be embarrassed to be seen as having an environmentally-friendly lifestyle (reverse-scored); To engage in recycling is an important part of who I am; I think of myself as a waste-conscious person. Responses were on a seven-point scale from Strongly disagree (1) to Strongly agree (7).

Contextual variables included demographic variables (Table 1) and the following.

- Recycling facilities: Do you have a recycling bin (or bins) in your laboratory? Yes (1), No (0), or Don't know (omitted from analysis). If yes, respondents were asked “Where is the nearest recycling bin positioned (in meters)?” Respondents were also asked: Do you have a doorstep recycling collection (e.g., green bin) where you live? and Did you have recycling facilities (e.g., green bins) where you went on your last holiday? with Yes (1), No (0), or Don't know (omitted from analysis) as response options.
- Waste policies and information: Two items measured workplace policies. These were: Does your organization have a policy to encourage recycling? Does your organization have a policy to encourage reuse of materials/equipment? Yes (1), No (0), or Don't know (omitted from analysis). A final question asked about information provision: Does your organization provide information on/near recycling bins about which materials can be recycled? Yes (1), No (0), or Don't know (omitted from analysis).

All means, standard deviations (SDs) and correlations are shown in Appendix 1 in Supplementary Material.

## Results

### Interviews

We outline here the main findings from the interviews, with exemplar quotes. All names reported are *pseudonyms* to protect interviewee confidentiality. Interview findings indicated (a) inconsistency between workplace contexts and between home and work; and (b) a range of barriers to and drivers of recycling in the workplace. In relation to the former, interviewees indicated that recycling is less common in labs than in offices, due for example to fewer recycling facilities in labs than in offices and more concern about contamination risks (see below). Furthermore, waste reduction at work more generally was less common than at home for various reasons, including not feeling responsible at work for dealing with waste:

“*At home I'm much more aware of it; I'll recycle everything I can. But here I shouldn't really say it, but there's just so much waste anyway, you don't feel as responsible for it I suppose. If I'm completely honest” –* Clara, Biosciences

Several others also noted a lack of responsibility for reducing waste. For example, Roger (Engineering) stressed that it is not something that can just be tacked onto somebody's workload; it would probably take up much of their time so would have to be a set role with sufficient time allocated. Likewise, Robin (Earth Sciences) concluded, “There's no accountability, that's the problem.”

Others admitted they (and colleagues) did not always recycle or reuse items because of the effort involved and availability of single-use items:

“*It's more convenient just to chuck it in the [general waste]. I must admit that we don't always put them through the recycling. It just becomes a matter of convenience”*—Roger, Engineering

“*Because there's always cups available, why would they do that [soak, rinse and dry them to reuse them]”—*Louise, Medicine

Indeed, this interviewee (Louise, Medicine) concluded that because of the effort involved in reducing waste, “I think you've really got to want to do it,” suggesting attitudinal factors (e.g., environmental values) might be important in the absence of a supportive context for waste reduction (see also “drivers,” discussed below).

Consistent with this, a variety of contextual (physical, organizational, informational) barriers to waste reduction were mentioned by interviewees. These included: unclear rules, lack of bin labeling, collection infrequency, limited storage space, limited awareness of facilities, health and safety regulations, actions by cleaners, and sterilization cost. In relation to health and safety rules, for example, Wendy (Earth Sciences) noted that she was limited in how many boxes she could keep for re-use as they posed a fire hazard. A common theme was a lack of recycling facilities; this included infrequent collection where facilities did exist:

“*[the sharps bins are] usually full, as you can see because all the broken glass is sort of propped on the top, which isn't very good”*—Johnny, Engineering

Concern was raised by three respondents (in two departments: Earth Sciences and Engineering) regarding rumors that cleaners tip recycling bins in with general waste, undermining individual efforts to sort waste:

“*There's always rumors that these things get chucked into the normal waste at the end of the day”*—Johnny, Engineering

“*Many people think they are recycling when in reality they're not. And it's not their fault … The fact that it's a blue bin doesn't mean anything to [the cleaners] […] I get it; the cleaning staff are busy, they're late, they've got tons of rooms to deal with. Having to deal with recycling and rubbish can be a bit of a burden”—*Robin, Earth Sciences

Lack of information about what can be recycled and where was also noted:

“*I'd be surprised if everyone in the building knows there's a recycling bin for these particular products down in the basement”—*Jared, Medicine

“*I think there's general confusion about how to recycle”—*Robin, Earth Sciences

The most commonly cited reason for not reusing or recycling items was risk of contamination (of both experiments and waste streams), mentioned by nine of the ten interviewees. In some cases, this led to a “blanket rule” that recycling bins were not permitted in labs (noted by Wendy, Earth Sciences) ostensibly to reduce contamination risk. In other cases, contamination risk was left to individual judgment and most adopted a precautionary approach:

“[The] *sterilization issue is the only reason why we wouldn't recycle.”—*Eileen, Biosciences

“*Unless you're absolutely certain that that vial is completely clean, it's very difficult to know whether you'd have contamination”*—Roger, Engineering.

“*The experiment has to come first, otherwise the results are meaningless”*—Johnny, Engineering.

Conversely, interviewees also mentioned some *drivers* of waste reduction. These included pragmatic factors, such as availability of supporting facilities or cost reduction. For example, several participants noted that some items could be reused at work by pooling equipment, where relevant schemes had been implemented. Others noted that “money is tight” (Robin, Earth Sciences), or the cost of buying new equipment instead of reusing items:

“*That's the big issue. People have no idea how much their tubes cost or how much the little cups cost… There's always a supply, but they have no idea how much these things cost.”—*Louise, Medicine

Other drivers of waste reduction were more normative or cultural, including personal values, habits (from home), social norms, and organizational policy or colleague encouragement. As the following quotes illustrate, waste reduction was viewed positively and normatively:

“*It's the right thing to do. There are moral issues with it—being wasteful when you don't have to be is wrong.”*—Robin, Earth Sciences

“*I just go on what you can recycle at home”*—Clara, Biosciences

“*If we're all doing it and we're encouraged to do it, it makes it happen”*—Louise, Medicine

“*We are an environmental lab so if we don't recycle who is going to recycle?”*—Eileen, Biosciences

The combination of these pragmatic and normative factors was identified by one interviewee:

“*It just makes sense, doesn't it? It's what we're supposed to do. It's the social thing isn't it. Partially I think. The thing to do now. Facilities are there, you're encouraged to take advantage of them, if you like”—*Jared, Medicine

### Survey

**Figure 1:**
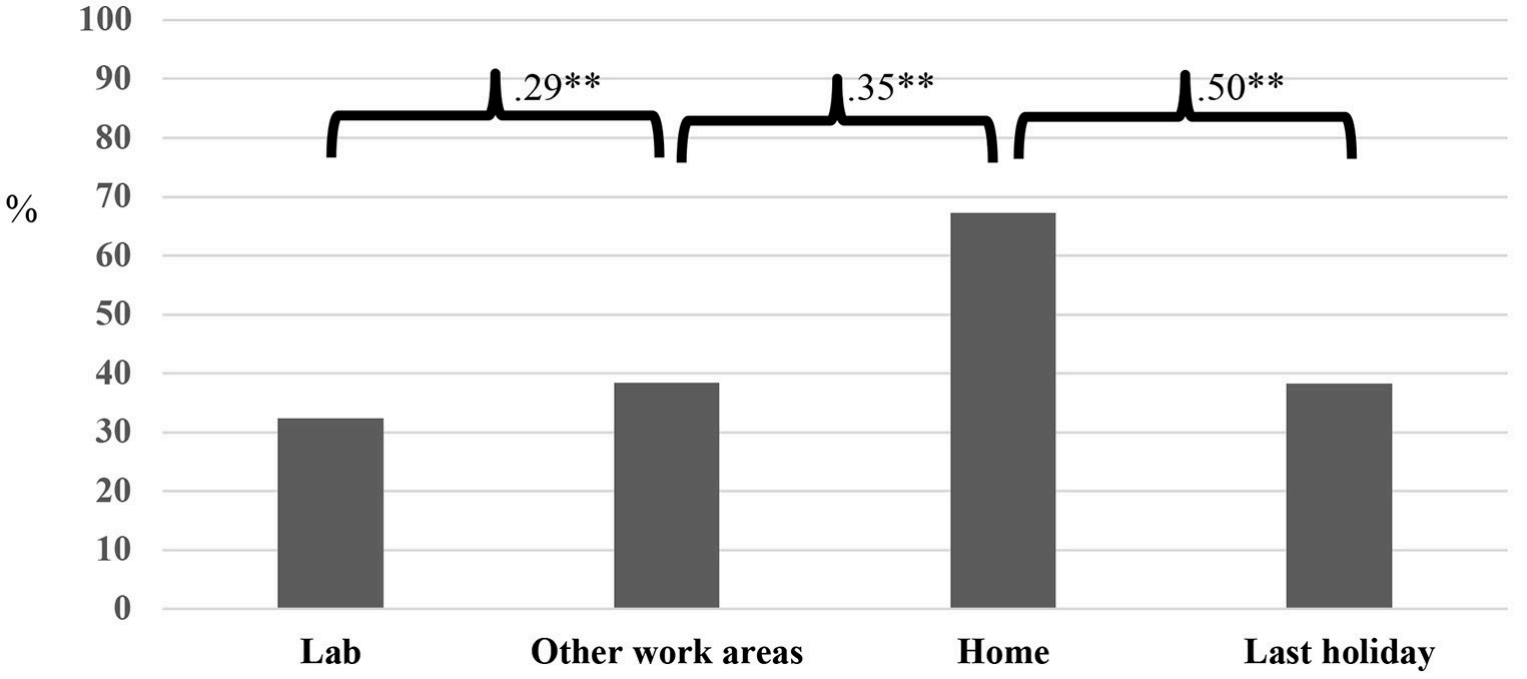
Proportion of waste recycled (% of total waste) across settings. ^**^Correlation is significant at 0.01 level (2-tailed).

**Figure 2:**
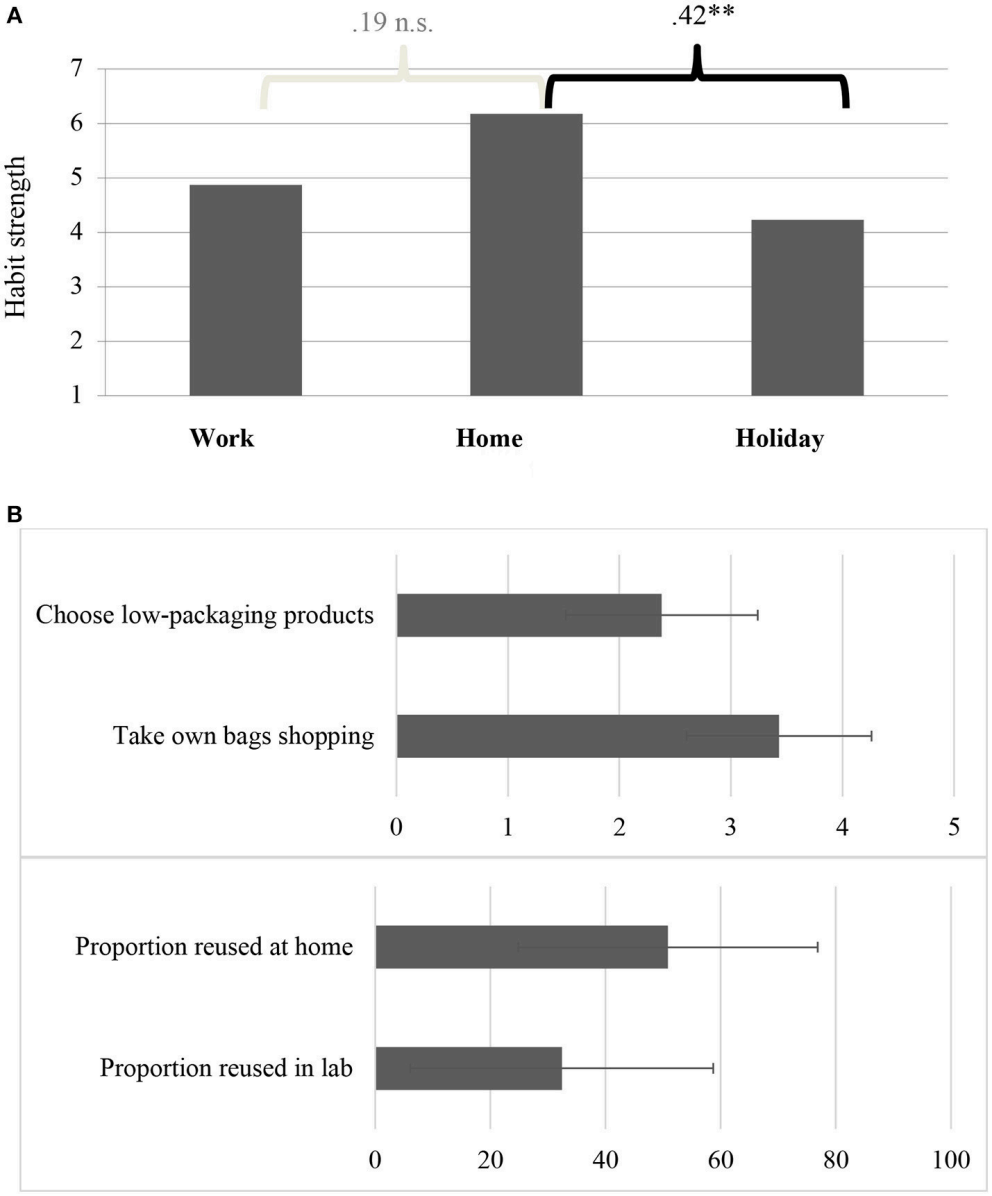
**(A**) Strength of recycling habit across settings (7-point scale). ^**^Correlation is significant at 0.01 level (2-tailed). **(B)** Reuse and reduction behaviors (domestic and workplace settings; top scale 0 = Never to 5 = Always, bottom scale %).

**Figure 3:**
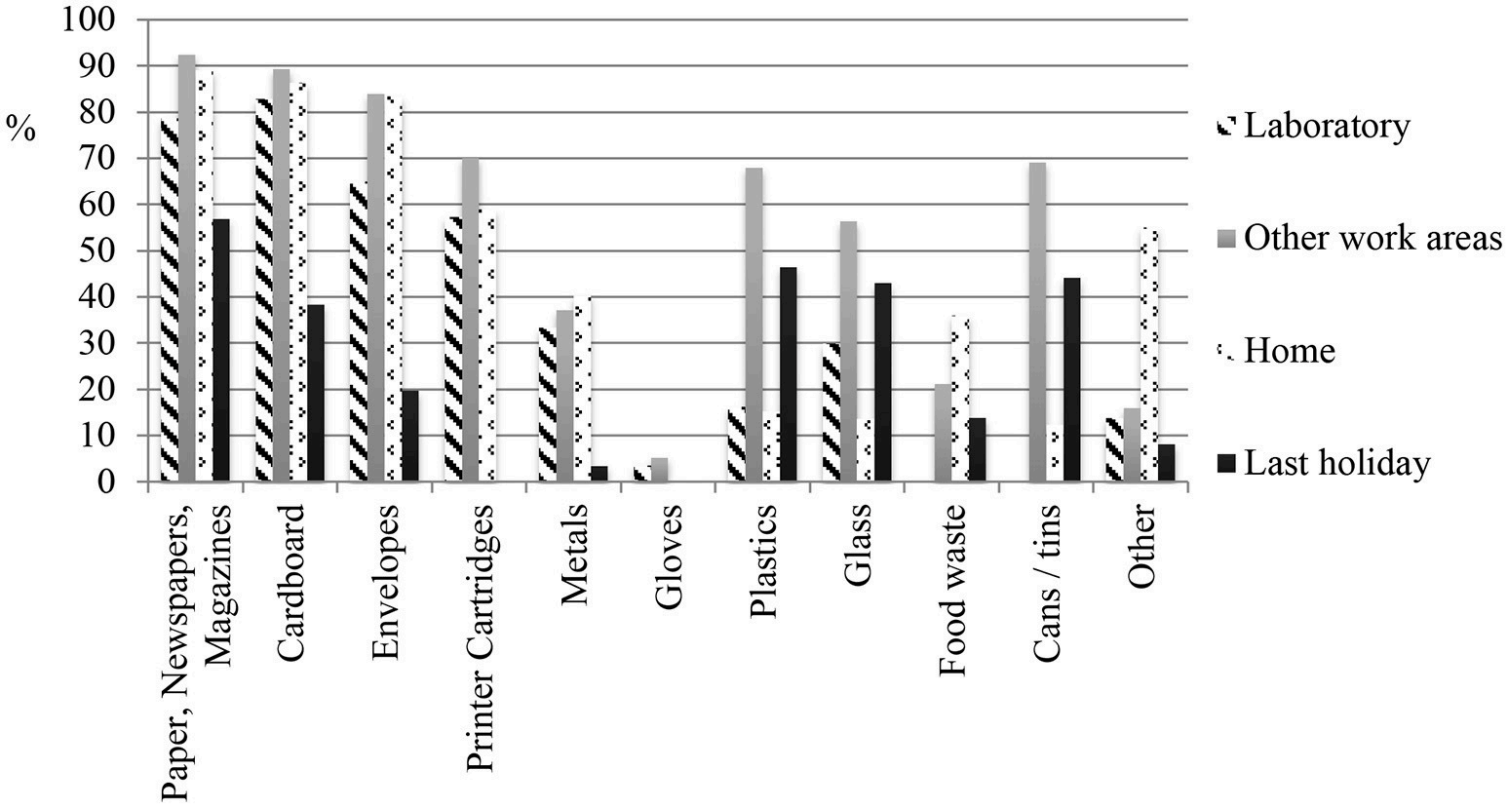
Percentage of different materials recycled across settings (there was no option to indicate that materials were not used at all).

As Figure 1 shows, the percentage of waste recycled at home, as estimated by participants (*M* = 67.3; *SD* = 19.1) is greater than in the workplace (Lab *M* = 32.4; *SD* = 26.3; Other work areas *M* = 38.4; *SD* = 25.1) and on their last holiday (*M* = 38.3; *SD* = 27.7). Consistent with this, the strength of recycling habits is higher at home than at work or on holiday (Figure 2A) and participants reuse a larger proportion of items at home than in the lab (Figure 2B). Furthermore, different materials are recycled in different locations, including within the workplace (laboratory vs. office; Figure 3).

**Figure 4:**
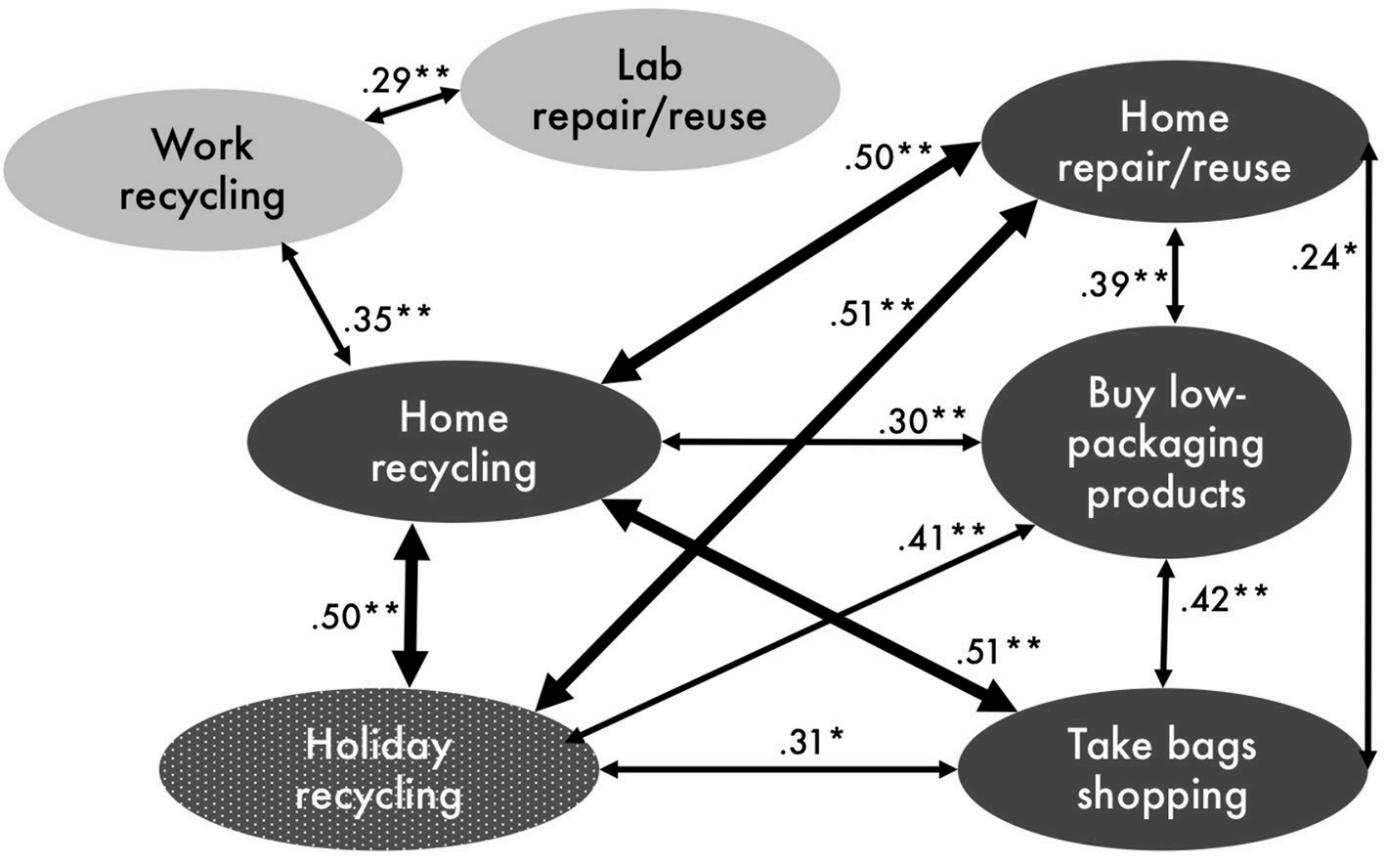
Correlations between waste reduction behaviors across contexts (thicker arrows indicate stronger correlations; dark balloons = domestic context; light balloons = work context; patterned balloon = holiday context). ^*^Correlation is significant at 0.05 level (2-tailed). ^**^Correlation is significant at 0.01 level (2-tailed).

Figure 4 shows the significant correlations between the behaviors measured within and across settings (see also Appendix 1 in Supplementary Material for non-significant correlations). Almost all waste behaviors are significantly correlated, although the strength of relationships varies considerably. Home recycling is significantly correlated with all other waste behaviors, both in the home and beyond it (apart from lab repair/reuse). Similarly, holiday recycling is related not only to domestic recycling but to all domestic waste behaviors. Conversely, workplace behaviors appear to be less related to behaviors in other contexts: workplace recycling is significantly co-related with domestic recycling, but not to any other behaviors; and lab reuse/repair is unrelated to behaviors outside the workplace (even to domestic repair/reuse).

We conducted step-wise regression analyses of recycling behavior across three contexts (lab, home, holiday), which enabled us to observe how much additional variance is explained over and above the TPB (model 1) when adding knowledge and contextual variables (model 2), and also identity and personal norm (model 3). As shown in Tables 2, 3, different, but overlapping, predictors are relevant in each setting. In laboratories, recycling is marginally predicted by attitude (model 1) and pro-environmental identity (model 3), while other predictors are non-significant. In the home, perceived behavioral control and knowledge are positive predictors, while attitude is a negative predictor in the full model. For holidays, perceived behavioral control, facilities, and personal norm are positive predictors. The results suggest that both contextual factors (e.g., facilities, PBC) and psychological factors (e.g., personal norm) are drivers of recycling behavior in different contexts, but also that different factors are important within each context. Our model of household recycling appeared to provide the best explanation of context-specific recycling of the three models, despite the additional explanatory variables included in our model of workplace recycling to anticipate differences between behavioral control in the workplace and other contexts.

**Table 2:** Predictors of recycling in the laboratory (% of waste recycled).

	**Beta**	***t***	***Model and R*^2^ (*R*^2^ change)**
(Constant)		−0.82	*1*
Attitude	**0.40**	2.06(^*^)	*0.20 (0.20)*
Social norm	0.17	0.90	
PBC	−0.05	−0.25	
(Constant)		−0.61	*2*
Attitude	0.28	1.23	*0.32 (0.12)*
Social norm	0.44	1.74	
PBC	0.06	0.26	
Knowledge	−0.38	−1.55	
Proximity of recycling bin	0.32	1.36	
Organizational recycling policy	−0.08	−0.31	
Info on recycling bin	0.24	0.93	
(Constant)		−1.52	*3*
Attitude	0.20	0.90	0.46 (0.14^*^)
Social norm	0.36	1.51	
PBC	−0.16	−0.65	
Knowledge	−0.23	−0.90	
Proximity of recycling bin	0.15	0.62	
Organizational recycling policy	0.07	0.28	
Info on recycling bin	−0.11	−0.38	
Pro-environmental identity	**0.49**	1.96(^*^)	
Personal norm	0.11	0.39	

* p < 0.1, ^*^p < 0.05.

*Significant values shown in bold*.

**Table 3:** Predictors of recycling at home and on last holiday.

	**Home—% recycled**			**Last holiday—% recycled**		
	**Beta**	***t***	***Model and*****R***^**2**^**(*****R***^**2**^**change)***	**Beta**	***t***	***Model and R**^**2**^**(R**^**2**^**change)***
(Constant)		0.40	*1*		−1.16	*1*
Attitude	−0.15	−1.61	0.42 (0.42^***^)	0.06	0.40	0.24 (0.24^**^)
Social norm	0.04	0.37		0.14	1.03	
PBC	**0.65**	6.49^***^		**0.42**	2.91^**^	
(Constant)		−0.02	*2*		−0.97	*2*
Attitude	**−0.23**	−2.57^*^	0.53 (0.11^***^)	−0.02	−0.14	0.35 (0.11^*^)
Social norm	−0.08	−0.82		0.11	0.84	
PBC	**0.45**	3.65^***^		**0.39**	2.67^*^	
Knowledge	**0.41**	3.95^***^			0.02	0.15
Recycling facilities	0.09	0.79		**0.34**	2.50^*^	
(Constant)		0.35	*3*		−0.66	*3*
Attitude	**−0.28**	−2.74^**^	*0.55 (0.02)*	−0.07	−0.40	0.41 (0.06^*^)
Social norm	−0.09	−0.92		0.11	0.81	
PBC	**0.46**	3.74^***^		**0.30**	2.04^*^	
Knowledge	**0.34**	3.13^**^		0.05	0.35	
Recycling facilities	0.07	0.64		**0.30**	2.28^*^	
Pro-environmental identity	−0.08	−0.87		−0.13	−0.67	
Personal norm	0.20	1.70		**0.35**	2.08^*^	

* *p < 0.05*,

** *p < 0.01*,

*** *p < 0.001*.

*Significant values shown in bold*.

Finally, consistency across contexts was explored by calculating an absolute difference score between the percentage of waste recycled at home and in the workplace (lab), and between home and their last holiday. This score was then used as a dependent variable in a linear regression with pro-environmental identity as predictor to determine to what extent pro-environmental identity explains cross-context consistency. This analysis found that consistency was not predicted by identity: (a) difference home-lab % recycled - identity *B* = 0.01, *p* = 0.96; (b) difference home-holiday % recycled - identity *B* = −0.18, *p* = 0.17.

## Discussion

### What Predicts Waste Behaviors in Different Contexts?

Our qualitative interviews showed that attitudes to recycling are largely positive, though there are barriers (e.g., lack of facilities/information, contamination risk) to translating intentions into action, as others have previously noted (e.g., Tudor et al., 2007). Indeed, the survey reinforces this finding, with contextual and control factors (recycling facilities, PBC) at least as important for predicting recycling as individual motivational or normative factors (e.g., identity, social norms). However, there were different predictors across contexts: Home recycling was predicted negatively by attitude, and positively by PBC and knowledge; Holiday recycling was predicted positively by PBC, recycling facilities, and personal norm; and work recycling was (marginally) positively predicted by pro-environmental identity. Overall, the TPB did not provide a sufficient explanation for recycling behavior in any location: social norms were not significant in any context, perhaps because recycling is now relatively normative, particularly amongst highly educated groups, such as the population we studied here (cf. Schultz et al., 1995; Thomas and Sharp, 2013). On the other hand, other non-TPB factors, such as recycling knowledge and personal norm, *were* found to be significant. The regression analysis shows attitude becomes a negative predictor when knowledge, PBC and recycling facilities were added to the equation. This negative role of attitude in home recycling is unexpected and difficult to explain. However, one possible explanation is that the inclusion of both knowledge and attitude creates an over-controlled model (Wooldridge, 2008). A prerequisite for such an explanation is met: that there is a moderate bivariate correlation between attitudes for home recycling and knowledge, *r* = 0.35, *p* < 0.01 (also PBC, *r* = 0.24, *p* < 0.05). Therefore, it is possible that the negative effect of attitude is a way in which, when controlling for the practical aspects—what, where and how to recycle—more abstract views about recycling do not always translate into recycling but the opposite (cf. De Young, 1989). Once variation in recycling due to recycling-knowledge is accounted for in the model, the remaining variation due to attitudes alone may represent only an abstract positivity toward the idea of recycling, and this abstract positivity may tend to increase to the extent that a participant does not actually engage with the reality of daily recycling. In addition, we found TPB variables account for much more variance at home (42%) than holiday (24%) or work (20%), perhaps because this context is more amenable to psychological factors such as those present in the TPB and other measured predictors (as suggested by the higher means for recycling attitudes, norms, PBC knowledge and recycling facilities at home than elsewhere; Appendix 1 in Supplementary Material). Indeed, we found few significant predictors of recycling at work, perhaps because there are strong institutional factors that impede the translation of TPB factors or other measured predictors into individual action by laboratory workers: such institutional factors are indicated by the interviews (e.g., health and safety regulations, cleaners' actions) but not all of these could be included in the survey due to space restrictions. Future research should therefore not assume TPB is equally valid across contexts and in particular should employ more organizational models (cf. Tudor et al., 2007) to explore workplace PEBs.

Our regression analyses also included variables not found in the TPB, which previous research indicated could improve upon a TPB explanation of waste-reduction behavior. Notably, we found personal norm to be a significant predictor of recycling on holiday, perhaps because motivation and ability to be pro-environmental on holiday tend to be lower than in everyday contexts (Barr et al., 2010; also Figure A1 in Supplementary Material) so for those people who do go to the effort of recycling on holiday they represent the most environmentally committed individuals. This is also consistent with the significant correlations observed between holiday recycling and all domestic waste reduction behaviors, suggesting those doing more waste reduction at home are the ones that take these habits on holiday. It would be interesting for future research to explore whether other models, such as the Value-Belief-Norm (VBN) model—which posits that personal norm is the proximal driver of pro-environmental action—would work better than TPB in certain contexts, such as on holiday.

### How Consistent Are People Across Waste Behaviors and Contexts?

Comparing prevalence of the same behaviors across contexts, we found that recycling at home is more common than in the workplace or on holiday; and similarly that repair/reuse at home is more common than workplace repair/reuse behaviors. This is consistent with the literature which indicates individuals tend to experience more barriers and/or less motivation to act pro-environmentally on holiday and at work than at home (e.g., Randles and Mander, 2009; Barr et al., 2010).

Consistent with expectations and the prior literature (e.g., Nash et al., 2017), we found more consistency (represented by significant, positive correlations) within contexts than between them. All domestic waste behaviors (recycling, reuse, reduce) were related; and both workplace behaviors (recycling, reuse) were related. Across contexts, the picture is more mixed: while recycling across the three contexts was significantly correlated, home and lab reuse behaviors were not. Holiday recycling, however, was significantly related to all domestic waste behaviors (not only recycling).

Together, these findings suggest there are more barriers to waste reduction (recycling and reuse) outside the domestic context than within it; and that contextual factors (e.g., facilities) are at least as predictive of waste reduction as individual factors, as indicated previously (Varotto and Spagnolli, 2017). At the same time as there being considerable variation across contexts, though, we also see heterogeneity across behaviors: recycling is more common than other waste reduction behaviors (consistent with other UK-based research, e.g., Whitmarsh et al., 2017) and apparently more transferable across contexts than repair/reuse behaviors. This may be because repair/reuse behaviors are potentially more diverse and dependent on context-specific requirements, skills and equipment (e.g., sterilization facilities in labs vs. kitchen sink at home; higher requirement for precision and cleanliness in lab than at home) than recycling behaviors, which require only a relevant receptacle (and information on what to put in it).

Given the relatively strong relationships between domestic recycling and most other waste behaviors, it is also interesting to speculate about whether recycling at home may be a “catalyst” behavior (Austin et al., 2011) to trigger subsequent waste reduction actions at home or elsewhere. Domestic recycling has been the focus of much environmental campaigning and of environmental psychological research for many years, and it is now widely practiced (Whitmarsh, 2009), but other waste reduction behaviors are less well-known and may be more difficult for individuals, due to structural constraints (e.g., use of excessive packaging by suppliers; Whitmarsh et al., 2017). Where policy measures have promoted these other behaviors, their adoption has increased, notably in the case of carrier bag reuse (Poortinga et al., 2013).

We tested whether pro-environmental identity was a significant predictor of cross-contextual consistency in recycling, and found that it was *not*. This is in contrast to most spillover models (e.g., Nash et al., 2017) and may indicate that contextual or other variables that prevent even the most motivated from acting on their identity are too strong an impediment in this case. Future work should explore other possible mediators for situational spillover, such as self-efficacy (Nash et al., 2017), behavioral control or use of similar materials/equipment which are indicated as being relevant in previous situational spillover research (Littleford et al., 2014).

### Implications and Limitations

The study highlights that both individual factors (e.g., pro-environmental identity) and contextual factors (e.g., facilities) are important in shaping individuals' waste behaviors; although different factors are more or less important in different contexts. Consistent with sociological perspectives on action (Schatzki, 2010), our results paint a picture of different drivers, constraints and “mindsets” (or social practices) occurring in different contexts. It may be that no single model (e.g., TPB) is able to adequately reflect this diversity. Similarly, the practical implication of these findings is that no single solution exists to improve waste reduction across diverse contexts, such as home, workplace and holiday settings. Indeed, there are also likely to be different measures required *within* each context to address different forms of waste reduction, including recycling, reuse and reduction behaviors. Recycling requires different forms of intervention or support (e.g., recycling bin, regular collection, information) than reuse or reduction behaviors (e.g., repair skills, storage space, product availability, changing norms around consumption; Whitmarsh et al., 2017).

This study adopted a mixed-method design, but did not undertake longitudinal or experimental analyses to ascertain causal pathways between behaviors. Similar to much previous “spillover” research (Nash et al., 2017), our correlational survey design only indicates relationships and consistencies across behaviors and contexts. Further work is needed to explore whether one behavior (e.g., home recycling) actually leads to adoption of further behaviors, and what factors mediate these behavioral or situational spillover processes. Our research also relied on self-reported recycling behavior, rather than observed recycling. Previous research shows these are positively correlated (Huffman et al., 2014) but there is generally a tendency to over-report pro-environmental behaviors due to social desirability (Kormos and Gifford, 2014), highlighting a need for future research in this area to include observational measures in addition to (or instead of) self-reports of recycling. Our measures could also be improved and expanded. For example, we asked about reuse of items in the home but there may be wide interpretations of what this applies to (e.g., crockery vs. packaging). More generally, there is a need for a greater range of reuse and reduction behaviors in future studies than we were able to include here, and to explore the range of determinants of these behaviors (as well as of recycling). We also note that our knowledge measure (particularly relating to the lab) had rather low reliability and could be improved in future work. Finally, our research focussed on one type of workplace (i.e., scientific research organizations), albeit including two very different contexts within that (laboratories and offices), with a UK-dominated sample. Future research should consider expanding cross-contextual spillover studies to other kinds of work environment (e.g., factories, shops, schools) and a wider range of cultures.

## Ethics Statement

This study was carried out in accordance with the recommendations of the British Psychological Society with written informed consent from all subjects. All subjects gave written informed consent in accordance with the Declaration of Helsinki. The protocol was approved by the School of Psychology Research Ethics Committee, Cardiff University.

## Author Contributions

LW designed the research, conducted the statistical analysis, and led the writing. PH assisted with statistical analysis and contributed to writing. MT undertook and analyzed the interviews, and contributed to writing.

### Conflict of Interest Statement

The authors declare that the research was conducted in the absence of any commercial or financial relationships that could be construed as a potential conflict of interest.
