# Supplementary material for: Waste Reduction Behaviors at Home, at Work, and on Holiday: What Influences Behavioral Consistency Across Contexts?
Source: Front Psychol. 2018 Dec 6;9:2447. doi: 10.3389/fpsyg.2018.02447 (PMC6291483; doi:10.3389/fpsyg.2018.02447)
Supplement: Supplementary file 1 [file Table_1.docx]

**Appendix 1. Means, SDs and Correlations of all variables**

**
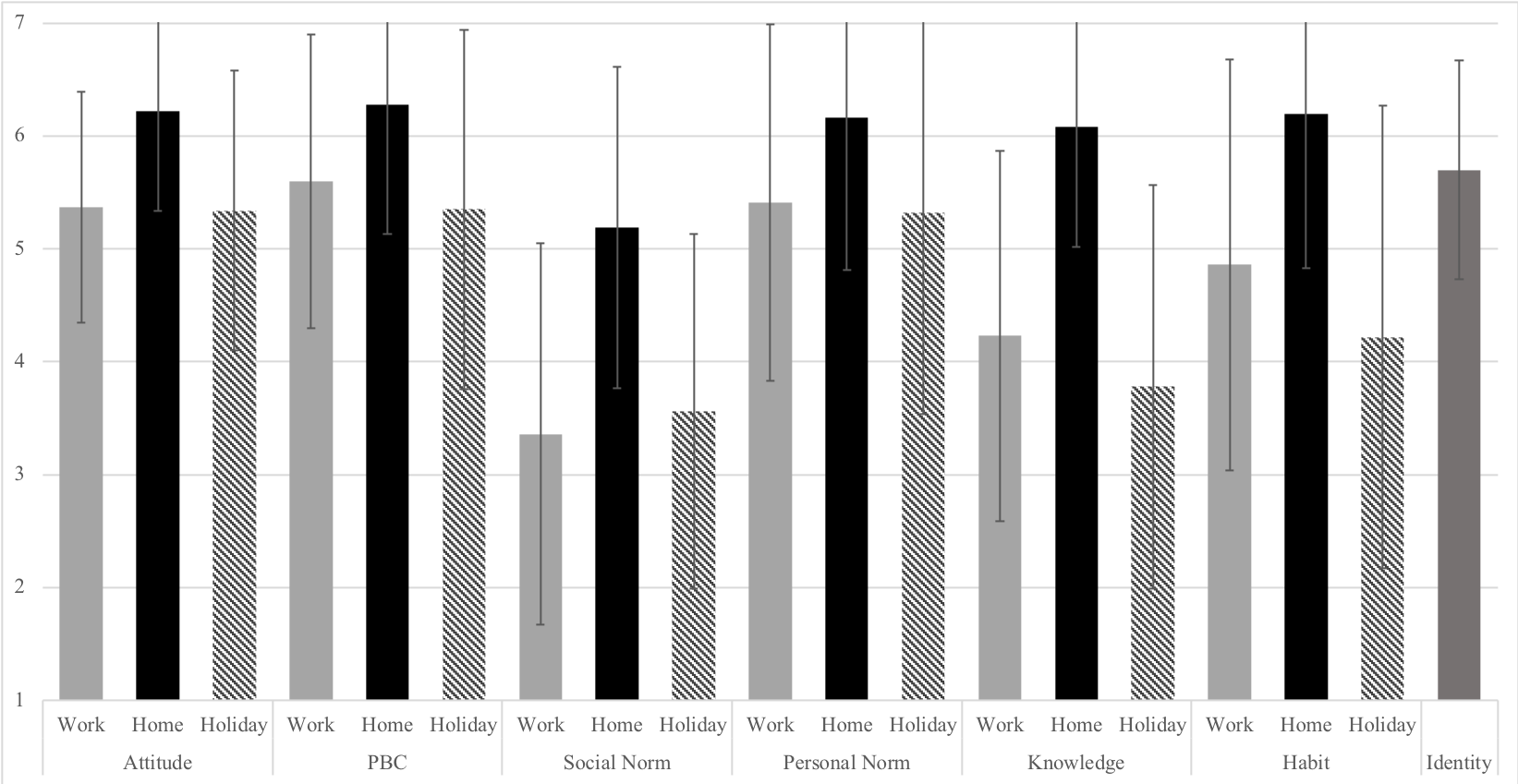
**

*Figure A1. Means and SDs of psychological variables*

*
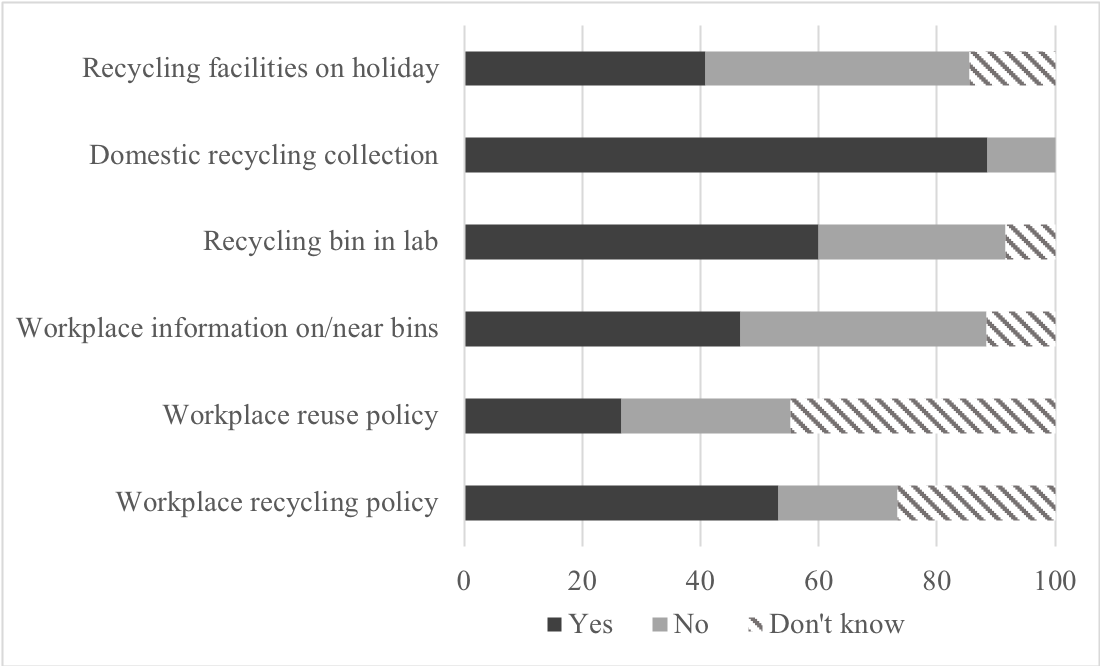
*

*Figure A2. Contextual variables (% of respondents)*
